# Optical Coherence Tomography of Animal Models of Retinitis Pigmentosa: From Animal Studies to Clinical Applications

**DOI:** 10.1155/2019/8276140

**Published:** 2019-10-30

**Authors:** Mitsuru Nakazawa, Aiko Hara, Sei-ichi Ishiguro

**Affiliations:** ^1^Department of Ophthalmology, Hirosaki University Graduate School of Medicine, Hirosaki, Japan; ^2^Department of Ophthalmology, Tohoku University Graduate School of Medicine, Sendai, Japan

## Abstract

**Purpose:**

The aim of this study was to understand the relationship between the findings of spectral-domain optical coherence tomography (SD-OCT) of previously reported animal models of retinitis pigmentosa (RP) associated with known genetic mutations and their background structural and functional changes.

**Methods:**

We reviewed previous publications reporting the SD-OCT findings of animal models of RP and summarized the characteristic findings of SD-OCT in nine different animal models (*RCS*^*–/–*^, *RHO* P23H, *RHO* S334ter, *RHO*^*–/–*^, *Rpe65*^*–/–*^, rp12, *Pde6β*^*–/–*^ (rd1 and rd10), and *Arr1*^*–/–*^) of human RP.

**Results:**

Despite the various abnormal structural changes found in these different animal models, progressive thinning of the outer nuclear layer (ONL) and hyperreflective change in the inner and outer segment (IS-OS) layers of the photoreceptors were commonly observed on SD-OCT. In the rapidly progressive severe photoreceptor degeneration seen in rd10 and *Arr1*^*–/–*^ mice, the ONL appeared hyperreflective. Electroretinography revealed various degrees of disease severity in these animal models. *Discussion and Conclusion*: SD-OCT is sensitive enough to detect even mild changes in the photoreceptor OS. Conversely, SD-OCT cannot qualitatively differentiate the pathologic and functional differences in the photoreceptors associated with different genetic abnormalities, with the exception of the rapid progression of severe forms of photoreceptor degeneration. These findings can be of value to understand better the clinical findings and the heterogeneous degenerative processes in patients with RP.

## 1. Introduction

**Figure 1:**
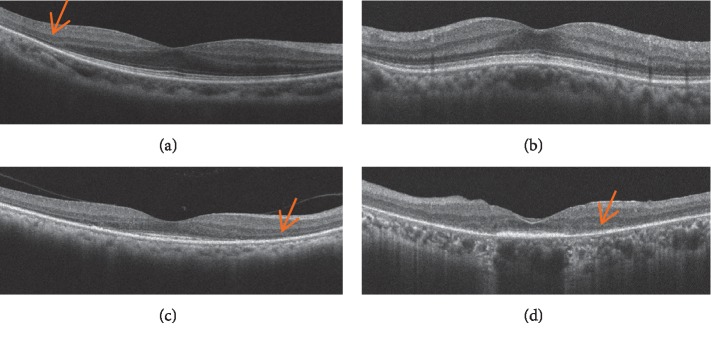
Phenotypic variability in the SD-OCT findings of patients with RP. (a) An SD-OCT image of a patient in whom the external limiting membrane, inner segment ellipsoid zone, and interdigitation zone (IZ) at the central macula appeared normal. (b) An SD-OCT image of a patient with a diffuse hyperreflective photoreceptor IS/OS layer. (c) An SD-OCT image of a patient in whom the IZ was absent. (d) An SD-OCT image of a patient with a diffusely hyperreflective short IS-OS layer. Arrows indicate the position of the outer nuclear layer.

Among the various conditions associated with inherited retinal degeneration, retinitis pigmentosa (RP) is the most common clinical entity; its prevalence is roughly one in 4,000–5,000 people worldwide [1, 2]. Typical clinical features include night blindness and photophobia beginning in the early stage, slow progression of constricted visual field loss and decreased visual acuity in the advanced stage, and eventual loss of vision in severe cases. Although most cases of RP are presumed to be monogenic, the condition is genetically heterogeneous and is known to be associated with mutations in more than 65 different genes (RetNet^TM^ (Retinal Information Network): https://sph.uth.edu/retnet/home.htm). In typical cases, RP is caused by photoreceptor cell death which primarily occurs in the rods, followed by cone cell death [1]. Because of the genetic heterogeneity of RP, the molecular mechanisms of photoreceptor cell death are also heterogeneous and include caspase-dependent apoptosis [3], calpain-related cell death [4–7], HDAK-related cell death [7], necrotic cone cell death [8], and autophagy [9]. These heterogeneous cell death mechanisms are derived from the extreme variety in the types and locations of mutations in different genes, which can presumably cause different metabolic deficits in the photoreceptors. Besides these different mechanisms of photoreceptor cell death, the difference in the causative mutations of RP is associated with various phenotypic features of photoreceptor degeneration that have mainly been investigated using animal models of RP [10–18]. Phenotypic heterogeneity is also observed in the clinical setting [1, 2]. Recent developments in the spectral-domain optical coherence tomography (SD-OCT) technology have provided valuable information regarding intraocular structure, especially in regard to pathologic changes in various vitreoretinal and chorioretinal diseases. In addition, SD-OCT has been applied to characterize RP by providing numerous structural parameters that help to analyze the correlation between various structures and the function, which has greatly expanded understanding about pathophysiology of RP [19–29]. Actually, in the clinical setting, various different SD-OCT findings are observed in patients with RP (Figure 1). Although OCT has enabled the noninvasive analysis of relationships between the structure and function in RP, the histopathological origins of the findings observed in SD-OCT images of RP are largely unknown because it is not possible to perform retinal biopsy in patients with RP. In addition, relationships between structural defects and functional impairments are yet to be found. To solve these problems, animal models of RP that harbor similar gene mutations to human RP may help to obtain clues to understanding the relationship between SD-OCT findings and the pathological background of RP and the associated functional deficits. We believe that the findings obtained from animal studies can be applied to both the quantitative and qualitative interpretation of SD-OCT images of patients with RP. In this review, we overviewed and summarized previous reports regarding the relationship between abnormal SD-OCT findings and their pathological origins using animal models of inherited retinal degeneration.

## 2. SD-OCT Findings in Mice, Rats, and Humans

**Figure 2:**
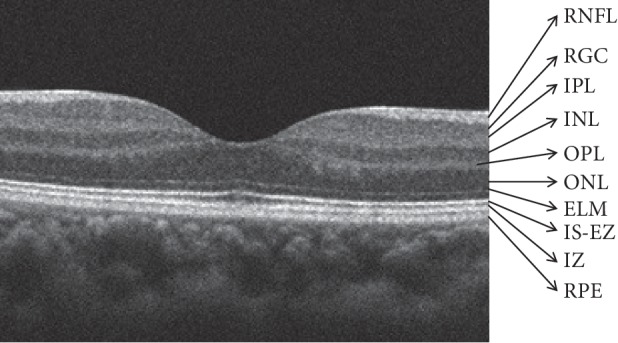
SD-OCT findings of the normal human macula. RNFL, retinal nerve fiber layer; RGC, retinal ganglion cells; IPL, inner plexiform layer; INL, inner nuclear layer; OPL, outer plexiform layer; ONL, outer nuclear layer; ELM, external limiting membrane; IS-EZ, inner segment ellipsoid zone; IZ, interdigitation zone; RPE, retinal pigment epithelium.

**Figure 3:**
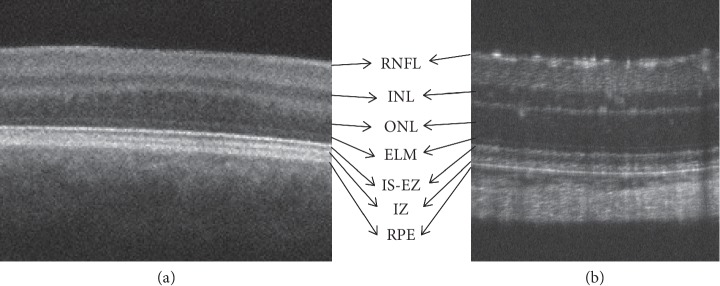
Comparison of the SD-OCT findings between a patient with macular hypoplasia and a wild-type rat. (a) An SD-OCT image of the central region of a patient with macular hypoplasia. (b) An SD-OCT image of a wild-type Sprague Dawley rat retina. RNFL, retinal nerve fiber layer; INL, inner nuclear layer; ONL, outer nuclear layer; ELM, external limiting membrane; IS-EZ, inner segment ellipsoid zone; IZ, interdigitation zone; RPE, retinal pigment epithelium.

**Figure 4:**
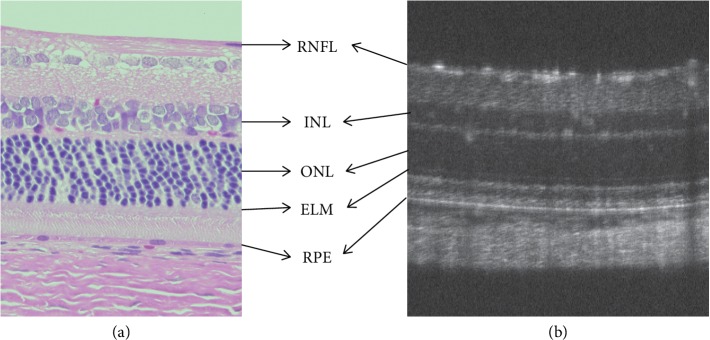
Comparison of the histological and SD-OCT findings of a wild-type Sprague Dawley rat. (a) Histological findings (hematoxylin and eosin staining). (b) An SD-OCT image of a wild-type Sprague Dawley rat retina. RNFL, retinal nerve fiber layer; INL, inner nuclear layer; ONL, outer nuclear layer; ELM, external limiting membrane; RPE, retinal pigment epithelium.

**Figure 5:**
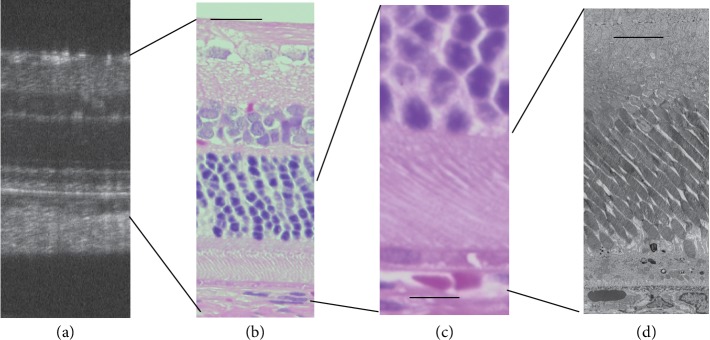
Comparison of the SD-OCT, histological, and electron microscopic findings in a wild-type Sprague Dawley (SD) rat. (a) An SD-OCT image of an SD rat on P33. (b, c) Histological findings (hematoxylin-eosin staining) of an SD rat on P65. (d) Electron microscopic examination of an SD rat on P65. Horizontal bars in (b–d) indicate 50 *μ*m, 20 *μ*m, and 10 *μ*m, respectively.

SD-OCT provides high-resolution visualization of the ocular structure [30–32]. In a normal human macular scan, an SD-OCT image of the retinal structure distinctly displays the retinal sublayers including the retinal nerve fiber layer (RNFL), retinal ganglion cell (RGC) layer, inner plexiform layer (IPL), inner nuclear layer (INL), outer plexiform layer (OPL), outer nuclear layer (ONL), external limiting membrane (ELM), inner segment (IS) ellipsoid zone (EZ), interdigitation zone (IZ), retinal pigment epithelium (RPE), and the choroid-like histological sections (Figure 2) [31, 32]. In contrast, because rats and mice do not have a macular structure, SD-OCT images of these animals appear similar to those of patients with foveal hypoplasia (Figure 3). Nevertheless, because cones are evenly distributed among rods even in the mouse retina (Supplemental Data 2 in [14]), SD-OCT images obtained from these animals are identical to the retinal sublayers in normal human subjects, as are the histological origins (Figure 4) and electron microscopic findings (Figure 5) [14, 33–35]. Based on the correlation between the SD-OCT imaging findings of rats and mice with retinal degeneration and their pathological and physiological features, these animals provide valuable models of the genotype-phenotype relationship in human RP.

## 3. Animal Models of RP and SD-OCT Analyses

### 3.1. Royal College of Surgeons (RCS^–/–^) Rats

*RCS* ^*–/–*^ rats are known to have unique pathological features in which photoreceptor degeneration occurs secondary to the defective phagocytic function of RPE [10]. Currently, the basic molecular deficit is known to be associated with homozygous mutations in the receptor tyrosine kinase, *mertk,* gene [36], which is specifically expressed in the RPE.

**Figure 6:**
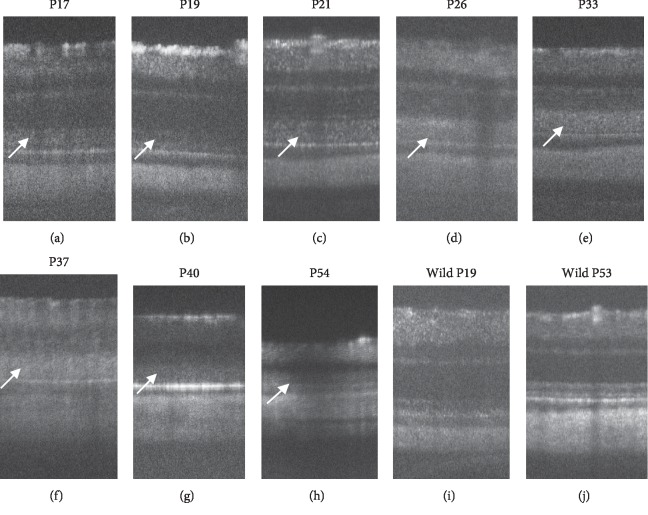
Longitudinal changes in the SD-OCT findings of *RCS*^–/–^ rats. (a–h) SD-OCT findings of the RCS^–/–^ rat retina from P17 to P54, respectively. (i, j) SD-OCT findings of wild-type RCS rats on P19 and P53, respectively. Arrows indicate diffuse hyperreflective changes in the photoreceptor inner and outer segment zones of the photoreceptors.

**Figure 7:**
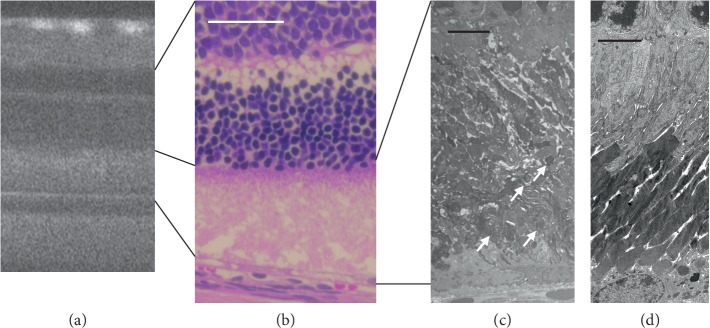
Comparison of the SD-OCT, histological, and electron microscopic findings of *RCS*^–/–^ rats. (a) An SD-OCT image of an RCS^–/–^ rat on P26. (b) The histological appearance of an RCS^–/–^ rat on P25, indicating degeneration in the inner and outer segment zones of the photoreceptors. (c) The electron microscopic findings of the photoreceptor inner and outer segment layers of an RCS^–/–^ rat on P25 demonstrated accumulation of the extracellular lamellar materials (arrows). (d) The same layer in a wild-type RCS rat. White bar indicates 50 *μ*m. Black bars indicate 5 *μ*m.

**Figure 8:**
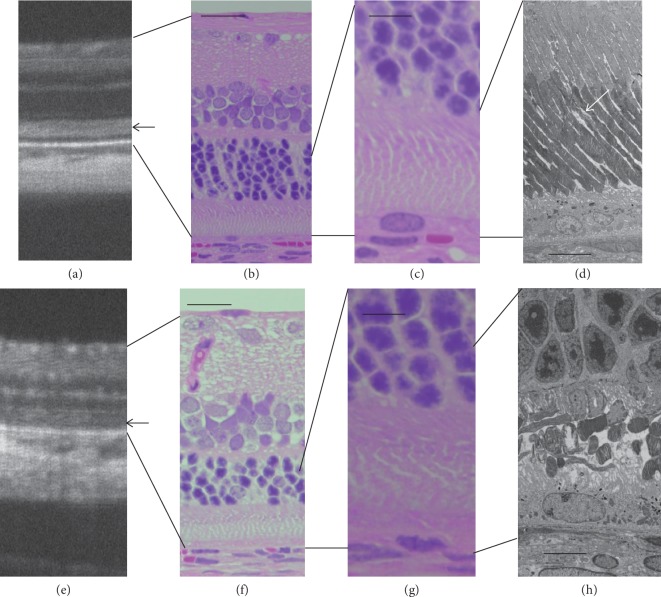
Comparison of the SD-OCT, histological, and electron microscopic findings of *RHO* P23H rats (line 2). (a, e) SD-OCT images of *RHO* P23H rats on P46 (a) and P125 (e), respectively. (b, f) Histological findings (low magnification) of *RHO* P23H rats on P62 (b) and P169 (f), respectively. (c, g) Histological findings (high magnification) of the photoreceptor layers of *RHO* P23H rats on P62 (b) and P169 (f), respectively. (d, h) Electron microscopic findings of the photoreceptor inner and outer layers of *RHO* P23H rats on P62 (b) and P169 (f), respectively. Note the different appearance of photoreceptor degeneration between panels (d) and (h), in spite of similar findings on SD-OCT ((a) and (c)). Bars in (b) and (f) indicate 50 *μ*m, those in (c) and (g) indicate 20 *μ*m, and those in (d) and (h) indicate 10 *μ*m. Black arrows indicate diffuse hyperreflective zones on SD-OCT images. A white arrow indicates disarrangement of the outer segment discs in a *P23H* rat on P62.

In addition, because mutations in *mertk* gene have been identified in patients with autosomal recessive RP [37], the retinal degeneration in the *RCS*^*–/–*^ rat can be considered as a model of human RP. The characteristic features of SD-OCT images during the progression of retinal degeneration in *RCS*^*–/–*^ rats have been reported by Adachi, Takahashi, and associates [34] and Ryals and associates [38]. The results of the two groups were quite similar. In the early stage of retinal degeneration, the IS-EZ lost its sharpness and gradually became broad after postnatal day (P) 17, while the photoreceptor IS and outer segment (OS) layer appeared diffusely hyperreflective after P21 (Figure 6) (Figure 3 in [34]; Figure 4 in [38]). Histological and electron microscopic analyses revealed that the hyperreflective OCT findings in the IS-OS layer corresponded to the progressive accumulation of extracellular lamellar materials, which appeared to be derived from debris of unphagocytized photoreceptor outer segments and degenerated photoreceptor IS and OS (Figure 7) (Figure 11 in [10]; Figure 6 in [34]). Because of the accumulation of the extracellular lamellar materials, the thickness of the IS-OS layer measured by SD-OCT was apparently maintained, even after P100 [34]. In addition, thickness of the ONL gradually began to decrease after P23 and completely disappeared after P53. These periods roughly correspond to the periods in which the calpain activity was reported to have increased in the retina [6]. Similar OCT findings were literally reported in patients with autosomal recessive RP associated with the deletion of exon 8 within the *mertk* gene [39], suggesting that the “hyperreflective bodies” observed in these patients might be derived from extracellular lamellar material-like unphagocytized degraded OS debris. Although the a-wave amplitude was reduced on electroretinography (ERG), the rate of reduction was faster than the rate of reduction of the thickness of the ONL (Figures 8 and 9 in [34]), suggesting that the a-wave amplitudes are influenced by the degeneration of both cell bodies and the IS-OS layer of the photoreceptor cells.

### 3.2. Rhodopsin (RHO) P23H Transgenic Rats

**Figure 9:**
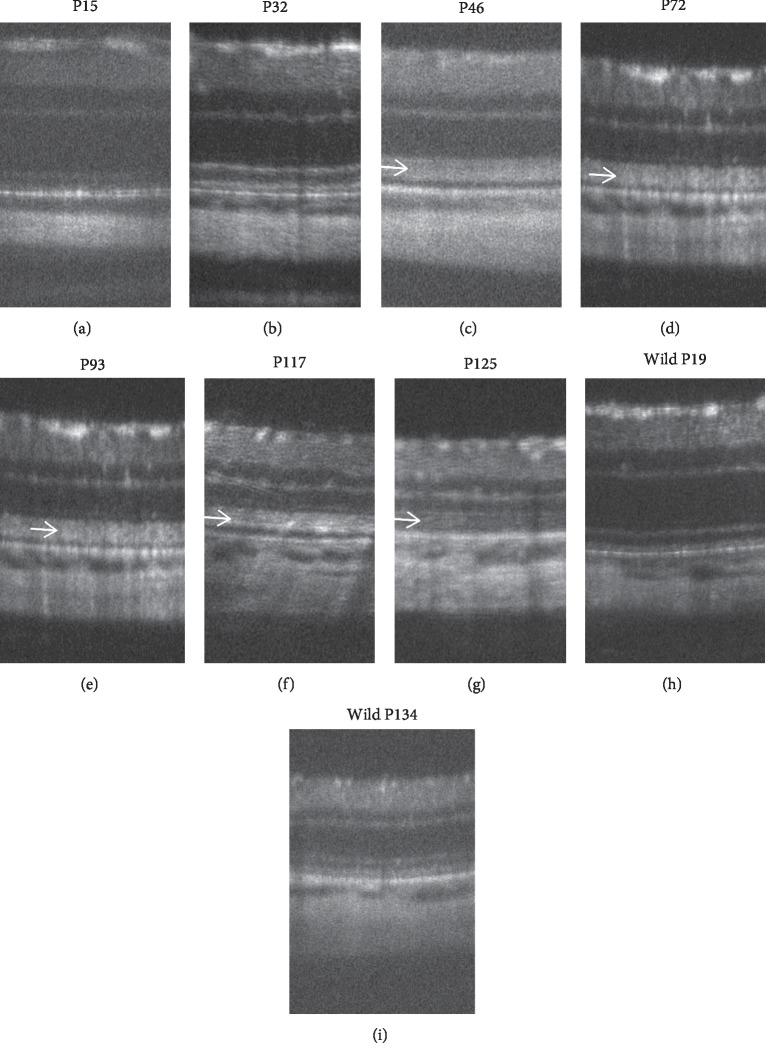
Longitudinal changes of the SD-OCT findings of *RHO* P23H rats. (a–g) SD-OCT findings of RHO P23H rat (line 2) retina from P15 to P125. (h, i) SD-OCT findings of wild-type SD rats on P19 and P134, respectively. Arrows indicate diffuse hyperreflective changes in the photoreceptor inner and outer segment zones.

A P23H point mutation in the rhodopsin (*RHO*) gene was first identified as a causative mutation for autosomal dominant RP and is known to be the most common mutation in RP patients in North America [40]. The *RHO* P23H transgenic rat was generated by introducing mouse *RHO* P23H transgenes into the oocyte of wild-type Sprague Dawley (SD) rats [41]. Because both the wild-type *RHO* gene and the P23H mutant *RHO* gene coexist in transgenic rats, *RHO* P23H transgenic rats can be considered as an animal model of autosomal dominant RP associated with heterozygous *RHO* P23H mutation. A point mutation in codon 23 of the *RHO* gene is classified as Class II, in which the mutant rhodopsin molecules result in misfolding during protein synthesis [18]. Most misfolded rhodopsin molecules cannot leave the endoplasmic reticulum (ER) and become subjected to ER-associated degradation (ERAD). Nevertheless, part of the misfolded rhodopsin molecules is not efficiently degraded by ERAD and accumulates in the ER, leading to ER stress on the photoreceptor cells [18]. Photoreceptor cell death is mainly triggered by ER stress [18] and the mechanisms of nonapoptotic caspase-independent cell death are reported to play a role [7]. In addition, part of the P23H mutant rhodopsin molecules can be transported to the Golgi apparatus and subsequently transferred through the connecting cilium and were incorporated into the OS disc membrane, although the mutant molecules lead to aberrant disc morphogenesis [42]. Thus, the OS discs are mostly composed of wild-type rhodopsin molecules and a small part of mutant rhodopsin molecules, resulting in the partial disarrangement of discs (Figure 8(d)) (Figure 13 in [17, 42]). The SD-OCT findings of *RHO* P23H transgenic rats on P29 of line 1 (severe type) [43] and the longitudinal changes from P15 to P287 of line 2 (mild type) have been reported [44]. The common findings between P23H lines 1 and 2 include diffuse hyperreflective change in the IS-OS layer associated with indistinguishable IS-EZ and IZ, and gradual thinning of the ONL (Figures 8 and 9) (Figure 6 in [43]; Figures 3 and 4 [44]). Although both changes are derived from progressive photoreceptor degeneration, even the partial disarrangement of the OS discs in the early phase results in the formation of the diffuse hyperreflective change of the IS/OS layer on SD-OCT, and SD-OCT cannot distinguish between the early change of the OS discs (P62 in line 2) and the severe disorganization of the discs in the late stage (P169 in line 2, Figures 8(d) and 8(h).

### 3.3. *RHO* S334ter Transgenic Rats

**Figure 10:**
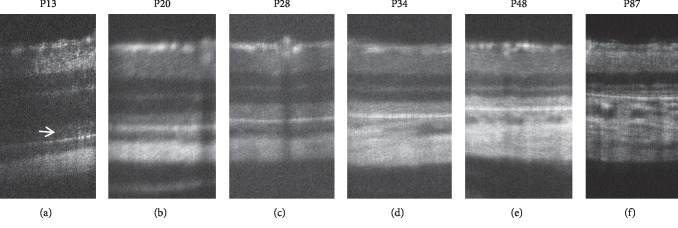
Longitudinal changes of the SD-OCT findings of *RHO* S334ter rats. (a–f) SD-OCT findings of the *RHO* S334ter rat retina from P13 to P87. Arrow indicates diffuse hyperreflective changes in the photoreceptor inner and outer segment zones.

**Figure 11:**
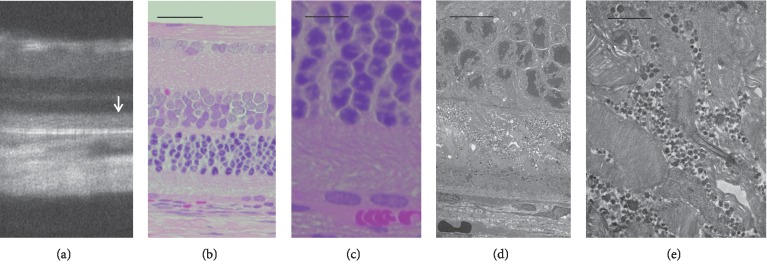
Comparison of the SD-OCT, histological, and electron microscopic findings of *RHO* S334ter rats (line 4). (a) An SD-OCT image of an *RHO* S334ter rat on P34. (b, c) Histological images (low and high magnifications) of *RHO* S334ter rat on P36, respectively. (d, e) Low and high magnifications of electron microscopic views of the same rat. Note the degeneration of the inner and outer segments of photoreceptors and the accumulations of a large amount of extracellular granular materials. Bars indicate 100 *μ*m in (b), 20 *μ*m in (c), 10 *μ*m in (d), and 2 *μ*m in (e).

**Figure 12:**
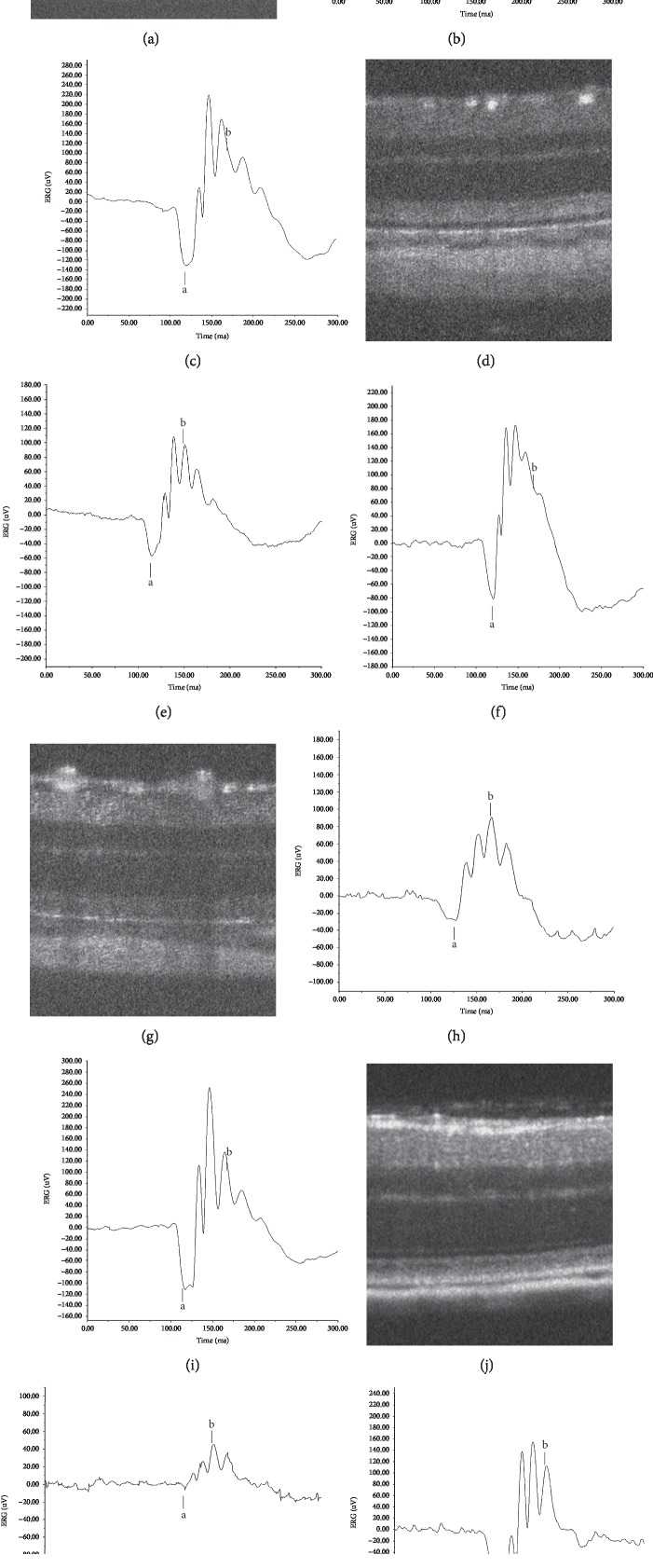
Typical ERG records when hyperreflective changes were first recognized on SD-OCT in four different genotypes. (a) An SD-OCT image of an RCS^*–/–*^ rat on P26. (b) An ERG record of an RCS^*–/–*^ rat on P28. (c) An ERG record of a wild-type RCS^*+/+*^ rat on P24. (d) An SD-OCT image of an RHO P23H rat on P46. (e) An ERG record of an RHO P23H rat on P42. (f) An ERG record of an SD rat on P46. (g) An SD-OCT image of an *RHO* S334ter rat on P15. (h) An ERG record of an *RHO* S334ter rat on P17. (i) An ERG record of an SD rat on P22. (j) An SD-OCT image of an RPE65^*–/–*^ mouse on P32. (k) An ERG record of an RPE65^*–/–*^ mouse on P31. (l) An ERG record of a wild-type C57BL/6J mouse on P35. The stimulus condition was 3.0 cd s/m^2^. Animals were dark-adapted for 24 h. The amplitudes of both the a- and b-waves are compared to those of wild-type mice in Table 1.

An S334ter point mutation in the *RHO* gene is classified as a Class I mutation, in which the translated rhodopsin molecule lacks the C-terminal end portion that normally includes the trafficking signal [18]. Consequently, the mutant rhodopsin S334ter molecules cannot be transferred to the OS and aggregate in the cytoplasm of the photoreceptor cell [45]. In addition to the degradation of the mutant protein molecules by the proteasome system, some of the mislocalized aggregated proteins are eliminated by secretion to the extracellular matrix to protect the photoreceptor cells [18]. However, if the aggregated protein molecules are accumulated over a certain limit, then they trigger the cell death mechanisms in a dose-dependent manner [18]. Namely, the *RHO* S334ter mutation induces various cell death pathways including caspase-dependent apoptosis and calpain-related nonapoptotic death and/or HDAC-PARP-related nonapoptotic death [7]. Generally speaking, the phenotypes associated with the Class I *RHO* mutations are more severe than those with Class II mutations [46]. The phenotype of transgenic rats associated with *RHO* S334ter is also more severe than that of P23H rats. The SD-OCT findings in the natural course of the retinal degeneration of *RHO* S334ter transgenic rats (line 4) were reported by Yamauchi and associates [47]. In their report, thinning of both the ONL and the IS-OS layer proceeded faster and more severely in comparison with P23H transgenic rats [43, 44], began as early as P13, and the diffuse hyperreflective zone also appeared in the IS-OS layer at the same time point (Figure 10) (Figure 1 in [47]). Electron microscopy revealed that the IS and OS were severely degraded on P23, and numerous tiny granule-like materials were distributed in the extracellular matrix even on P23 (Figure 11) (Figure 3 in [47]). These tiny granules probably contain the secreted aggregations of mutant rhodopsin S334ter molecules. It has also been suggested that the diffuse hyperreflective zone originates from both the degenerated IS and OS and extracellularly distributed granules [47]. However, it is impossible to qualitatively differentiate the diffuse hyperreflective zone on SD-OCT caused by S334ter from that caused by P23H. The reduction of the amplitudes of both the a- and b-waves on ERG also progressed faster in S334ter rats than in P23H rats (Figure 12) (Figures 5 and 6 in [47]).

### 3.4. RHO^–/–^ Mice

*RHO* ^*–/–-*^ mice were created by disruption of the mouse *RHO* gene [48]. In *RHO*^*–/–*^ mice, rhodopsin is not expressed in the photoreceptor cells and the rod OS is not formed [49]. In addition to the absence of the rod OSs, a gradual decrease in the number of photoreceptor cells was confirmed by histological observation [33, 34, 50, 51]. The photoreceptor cell death is reportedly processed by the calpain-related nonapoptotic death and/or HDAC-PARP-related nonapoptotic death [7]. In SD-OCT, in addition to the gradual decrease in thickness of the ONL, no EZ and IZ were observed, although a thin hyporeflective layer was detected below the ELM [33, 50–52]. Histologically, although the OS was not developed, the IS-like structure was found [50, 51]. Thus, the hyporeflective layer was considered to be originated from the IS-like structure.

### 3.5. Rpe65^–/–^ and rd12 Mice

RPE65 (retinal pigment epithelium-specific 65-kDa protein) is an enzyme that is specifically expressed in the RPE and catalyzes the isomerohydration of all-*trans*-retinyl ester to 11-*cis*-retinol [53]. It plays a critical role in the reaction through which chromophores are recycled (visual cycle; i.e., all-*trans*-retinal to 11-*cis*-retinal). Defects of RPE65 have been known to cause a type of autosomal recessive RP or Leber congenital amaurosis (LCA), which is a type of early-onset RP that occurs in children [54–58]. Thus, mutations in the *Rpe65* gene lead to malfunction in the supply of 11-*cis*-retinal to the photoreceptor cells and the accumulation of all-*trans*-retinyl ester in the RPE and subsequently induce the consecutive degeneration of photoreceptor cells. It was previously reported that the disruption of the mouse *Rpe65* gene (*Rpe65*^–/–^ mice) caused molecular instability and mistrafficking of both the S- and M-cone visual pigments from the IS to the OS, whereas apo-rhodopsin (rhodopsin without chromophore) was normally incorporated into the OS discs, although the size of the rod OS gradually decreased [11, 14]. The photoreceptor cells in the *Rpe65*^–/–^ mice showed a caspase-independent cell death reaction [7]. Based on these molecular mechanisms, it is likely that the retinal degeneration in the *Rpe65*^–/–^ mice demonstrated slow rod degeneration and rapid cone degeneration [11, 13, 59], which mimicked the early stage of human LCA [60]. In addition, because the genetic defect of the *Rpe65*^–/–^ mice is RPE-specific and not photoreceptor-specific, supplementation of the intact *Rpe65* gene to the RPE of the *Rpe65*^–/–^ animals improved their photoreceptor function [61–68]. However, dog experiments demonstrated that the gene therapy performed in early age seemed to be more effective in comparison with gene therapy performed in adult age [64]. These results suggested that the progression of the retinal degeneration was biphasic: (1) in the early stage, the photoreceptor cells keep reversibility, to some extent, and (2) they become nearly irreversible in the later age in the dog associated with *Rpe65* gene mutations.

**Figure 13:**
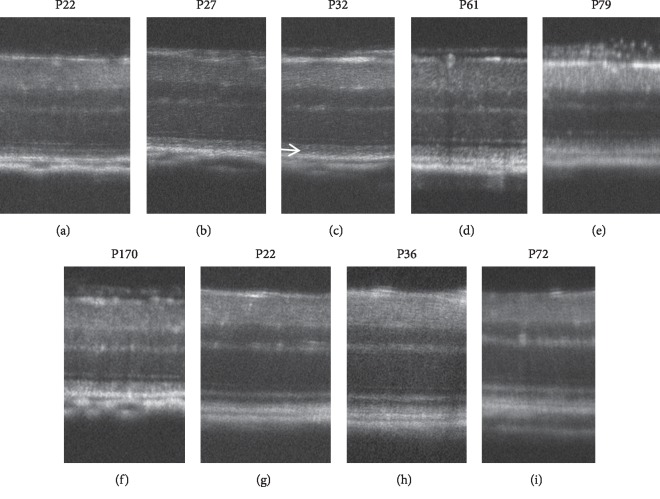
Longitudinal changes in the SD-OCT findings of *Rpe65*^–/–^ mice. Longitudinal changes in the SD-OCT findings of *RPE65*^/–^ mice. (a–f) SD-OCT findings of *RPE65*^–/–^ mice retina from P22 to P170. Arrow indicates diffuse hyperreflective changes in the photoreceptor inner and outer segment zones. (g–i) SD-OCT findings of wild-type C57BL/6J mice.

**Figure 14:**
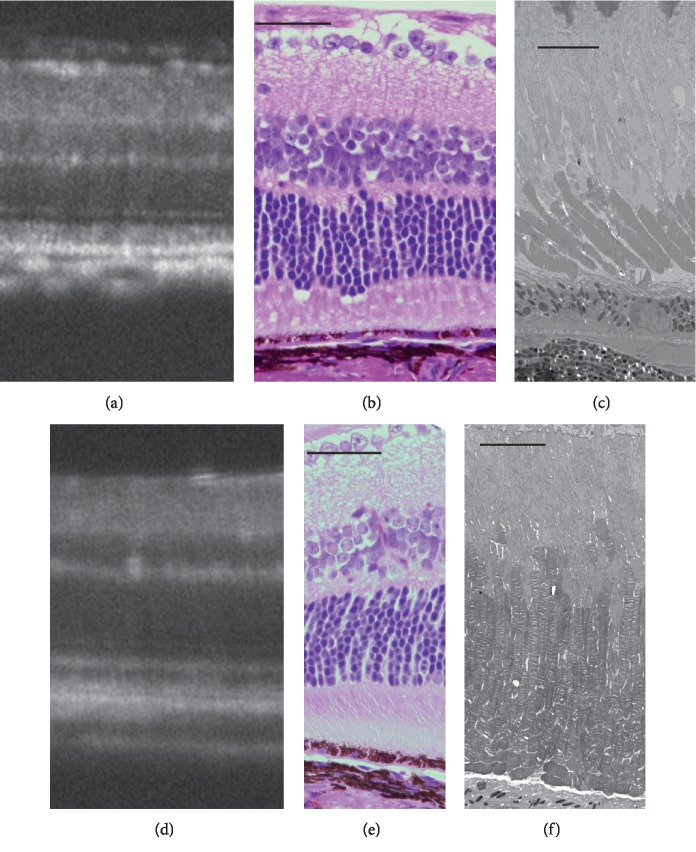
Comparison of the SD-OCT, histological, and electron microscopic findings of *RPE65*^–/–^ mice. (a) An SD-OCT image of an *RPE65*^–/–^ mouse with a diffuse hyperreflective zone in the inner and outer segment layers on P170. (b) The histological appearance of an *RPE65*^–/–^ mouse on P127. (c) The electron microscopic findings of an *RPE65*^–/–^ mouse on P127. Note the short-sized photoreceptor outer segments with regularly packed discs. (d) An SD-OCT image of a wild-type C57BL/6J mouse on P72. (e) The histological appearance of a wild-type C57BL/6J mouse on P66. (f) The electron microscopic findings of a wild-type C57BL/6J on P66. Bars indicate 100 *μ*m in (b) and (e) and 10 *μ*m in (c) and (f), respectively.

The SD-OCT findings of the natural course of retinal degeneration in *Rpe65*^–/–^ mice have been reported by Tanabu and associates [35]. Qualitatively, the IS-OS layer continuously appeared to be diffusely hyperreflective in *Rpe65*^–/–^ mice after P32 (Figure 13). In a quantitative study, the ONL in *Rpe65*^–/–^ mice appeared progressively thinner in comparison with the ONL of the wild-type mice. In addition, although the IS-OS layer in *Rpe65*^–/–^ mice also becomes thinner in comparison with wild-type mice throughout the observation periods (P22 through P170), the thickness of the IS-OS layer plateaued from P36 to P170 (Figure 5 in [35]). On electron microscopy, the outer segments of the photoreceptors appeared severely degenerated during the early stage (∼P35), whereas the OS discs subsequently seemed less degenerated but were generally short in the late stage (from P49 to P127) (Figure 14) (Figure 4 in [35]). These results indicated that both the degeneration of the OS in the early phase and the randomly shortened but mildly disorganized OS in the late phase commonly resulted in the diffuse hyperreflective zone of the IS-OS layer on SD-OCT. In addition, although there were differences in the electron microscopic findings, SD-OCT could not qualitatively differentiate these findings. Scotopic ERG (3.0 cd s/m^2^) showed severe deterioration, even at P31 (Figure 12) (Figures 6 and 7 in [35]).

The rd12 mouse, which has a nonsense mutation in the exon 3 of the *Rpe65* gene (R44ter), is another mouse model of retinal degeneration caused by a mutation of the *Rpe65* gene [64]. Because of the R44ter mutation, RPE65 is not expressed in the RPE in homozygous rd12 mice. Like *Rpe65*^–/–^ mice, rhodopsin conjugated with chromophore is not detected in the retina of homozygous rd12 mice [11, 65]. The SD-OCT findings of rd12 mice are similar to those of *Rpe65*^–/–^ mice. The ONL gradually loses its thickness and the IS-OS layer becomes diffusely hyperreflective, as have been confirmed by SD-OCT in rd12 mice at P21 and 13 months (Figure 1 in [66]). Electron microscopy revealed a slightly disorganized OS, intracellular vacuoles in the ONL, and intracellular lipid vacuoles in the RPE in 7-month-old mice, whereas severe disorganization of the OS became obvious in 19-month-old mice (Figure 5 in [67]). Hasegawa and associates considered the intracellular vacuoles in the ONL to be autophagosomes [66]. It is suggested that although SD-OCT can detect structural abnormality in the IS-OS layer as diffuse hyperreflective changes, it cannot qualitatively differentiate the microstructural differences between the mild early and severe late phases. Scotopic ERG (3.0 cd s/m^2^) showed severe deterioration, even at P21 [66]. The results of the ERG studies of *Rpe65*^–/–^ and rd12 mice suggest that a defective supply of 11-*cis*-retinal and subsequent structural abnormality of the OS contribute to the deterioration of both the a- and b-waves.

### 3.6. Pde6*β*^–/–^ Mice (rd1 and rd10)

Phosphodiesterase 6 (pde6) is an enzyme that is activated by the activated transducing, which catalyzes conversion from cyclic GMP (cGMP) to 5′-GMP in the OS. This reaction is part of the phototransduction cascade, which leads to the subsequent closure of the cGMP-gated cation channel in response to light. Defects of the *β*-subunit of pde6 (pbe6*β*) interrupt the phototransduction cascade by preventing closure of the cGMP-gated cation channel, leading to constitutive hyperpolarization of the photoreceptor cells. These reactions subsequently trigger photoreceptor cell death [7] and finally cause RP [67]. Thus, mice lacking the intact pde6 *β*-subunit associated with mutations in the *Pde6β* gene (rd1 and rd10 mice) can be considered as mouse models of RP. Y347ter nonsense and R560C missense mutations in the *Pde*6*β* gene are detected as genomic abnormalities in rd1 and rd10, respectively [68, 69]. The SD-OCT findings of retinal degeneration in rd10 have previously been reported [60, 66, 70, 71]. Longitudinal observation of the ONL by SD-OCT revealed that the ONL transiently became hyperreflective at P20-21 and that the progressive thinning of the ONL was observed after P21 [66, 70]. In addition, intracellular vacuoles were observed in the ONL of rd10 at P21 [68]. Hyperreflective change in the ONL has never been detected in the other above-discussed other animal models of hereditary retinal degeneration. As discussed later, in *Arr1*^*–/–*^ mice, hyperreflective change of the ONL may indicate that the photoreceptor cells are subject to an acute cell death reaction [66]. The ERG study indicated that both a- and b-waves were almost extinguished by the stimulation of scotopic 3.0 cd s/m^2^ at P28 [60], suggesting that the photoreceptors are severely degenerated by P28.

In rd1, which shows a severe form of retinal degeneration, the ONL became extremely thin, even at P14, and could not be detected after P14, although the inner retinal layers were well preserved until at least P34 [33, 52, 70].

### 3.7. Arrestin 1^–/–^ Mice

Arrestin 1 is a protein that quenches photoactivated rhodopsin by competitively binding to phosphorylated rhodopsin against transducin. It plays an important role in the recycling of the rhodopsin molecule. Previous studies have revealed that mutations in the *Arrestin 1* gene (*Arr 1*, also abbreviated as *SAG*, soluble antigen) cause Oguchi disease, a type of autosomal recessive “stationary” night blindness [72] or autosomal recessive RP [73]. Recently, Nishiguchi and associates reported that patients with *Arr 1* (*SAG*) gene mutations show phenotypic heterogeneity varying from stationary Oguchi type to progressive RP type [74]. Thus, the *Arr1*^*–/–*^ mice can be considered to be a mouse model of RP [75]. SD-OCT of *Arr1*^*–/–*^ mice with light-induced acute retinal degeneration revealed striking findings after 12 h to 36 h of constant 200 lux illumination [75]. In the *Arr1*^*–/–*^ mice, after 12 h of continuous light exposure, in addition to the diffusely hyperreflective change of the IS-OS layer associated with obscuration of the IS-EZ, the ONL was thinner in comparison with wild-type mice. Furthermore, after 36 h of illumination, not only the IS-OS layer but also the ONL became diffusely hyperreflective (Figure 2 in [75]). The electron microscopic features of the retinal degeneration revealed IS vesiculation, severe disruption of the IS-OS junction, and condensation of the photoreceptor nuclei after 12 to 24 h of constant light exposure (Figure 3 in [75]). These acute pathological processes of the photoreceptor cell body in the light-induced *Arr1*^*–/–*^ mice correspond to the diffuse hyperreflective appearance of the ONL in the SD-OCT. Conversely, photoreceptor cell death occurred in other RP models (e.g., *RCS*^*–/–*^, *RHO* P23H, *RHO* S334ter, *Rpe65*^*–/–*^, and rd10) did not demonstrate any hyperreflective change in the ONL, while rd12 did. These differences in SD-OCT findings may represent differences in the severity or velocity of the cell death processes between them. Clinically, focal hyperreflective changes of the ONL can been seen in presumed acute ischemic lesions of photoreceptor cells, such as paracentral acute middle maculopathy (PAMM) [76]. It is suggested that the acute pathological changes in the ONL seen in these experimental (*Arr1*^*–/–*^) and clinical (PAMM) examples commonly result in the hyperreflective change in the ONL seen on SD-OCT.

## 4. Variability of the Structure-Function Relationship

The relationships between structure, as evaluated by longitudinal SD-OCT imaging, and the function, as characterized by ERG (dark-adapted single-flash ERG stimulated at 3.0 cd s/m^2^), have been reported in *RCS*^–/–^ rats [34], *RHO* P23H-2 rats [44], *RHO* S334ter-4 rats [47], and *Rpe65*^–/–^ mice [35]. In these animal models, the common pathological features observed on SD-OCT include diffuse hyperreflective change of the IS-OS layer and thinning of the ONL, although there were differences in the time points of their appearance and electron microscopic features. Because of the difference in their severity, the ERG patterns were also variable. Figure 12 and Table 1 show age-matched ERG records along with SD-OCT images obtained when the diffuse hyperreflective zone was first detected in each model animal. Although the SD-OCT findings of the IS-OS layer and ONL appear similar in these models, the amplitudes of both the a- and b-waves vary according to the types of mutations. It is suggested that these examples of phenotypic heterogeneity are based on their genetic heterogeneity, which leads to different cell death pathways [7].

## 5. Conclusion

In this study, we reviewed and analyzed the previous reports on the SD-OCT imaging findings in animal models of RP.  Table 2 summarizes the data described in this review. The aim of this study was to comprehensively understand the pathological origins of the abnormal SD-OCT findings in animal models of RP and to identify characteristic findings associated with the death of certain types of photoreceptor cells in RP models. Conversely, the results showed that diffuse hyperreflective change of the IS-OS layer and thinning of the ONL were relatively common SD-OCT changes in six different genotypes (*RCS*^*–/–*^ (*mertk*^–/–^), *RHO* P23H, *RHO* S334ter, *Rpe65*^–/–^, rd12, and *pde6β*^*rd10*^). Although these six genotypes presented unique pathological features on electron microscopy, which depended on the types of mutation, these differences were not seen on SD-OCT. These results suggest that the optical sensitivity of SD-OCT is sufficient to detect even minor changes in the IS-OS layer and present such changes as hyperreflective change, irrespective of the pathological severity. The diffuse hyperreflective change in the IS-OS layer on SD-OCT in patients with RP may be derived from IS-OS degeneration of various degrees of severity, ranging from mild partial disarrangement of the OS discs to severe deterioration of the IS-OS. Conversely, because of the high sensitivity of SD-OCT, when SD-OCT images show a normal macular appearance in patients with RP, histological examinations and electron microscopic may show no abnormal changes in the macular area in these patients. Similarly, even if the SD-OCT findings are similar in patients with RP, their severity—as assessed by ERG—may vary due to the heterogeneity of the pathological conditions underlying the SD-OCT findings. Regarding the ONL findings, thinning of the ONL is commonly seen among various animal models. The only exception was that the hyperreflective change in the ONL was observed in the rd10 at P20-21 and light-stimulated *Arr1*^–/–^ mice. The histological analysis of these mice suggests that this change may be derived from the severe and rapid degeneration of the photoreceptor cell bodies, which may differ from the relatively slow progression of photoreceptor degeneration in other animal models of RP.

The information summarized in this review can be of value to understand better the clinical findings of RP, particularly when considering the pathological changes underlying the SD-OCT findings in patients with RP.

## Figures and Tables

**Table 1:** Amplitudes of a- and b-waves of animal models of retinitis pigmentosa in Figure 12.

Animal models	Postnatal day	a-wave, *μ*V (% of wild-type)	b-wave, *μ*V (% of wild-type)
RCS^+/+^ rat	P24	102.01 ± 28.75	182.30 ± 65.43
RCS^–/–^ rat	P28	16.41 ± 1.69^*∗∗*^ (16.09)	78.70 ± 4.95^*∗*^ (43.17)
SD rat	P46	98.000 ± 16.971	279.500 ± 34.477
RHO P23H-2 rat	P42	43.712 ± 15.558^*∗*^ (44.60)	155.022 ± 30.272^*∗*^ (55.46)
SD rat	P22	89.848 ± 16.933	281.515 ± 51.096
*RHO* S334ter-4 rat	P17	25.566 ± 2.732^*∗∗*^ (28.45)	97.607 ± 12.585^*∗∗*^ (34.67)
C57BL/6J mouse	P35	38.33 ± 4.18	112.17 ± 23.08
RPE65^–/–^ mouse	P31	5.63 ± 0.375^*∗∗∗*^ (14.69)	58.43 ± 11.45 (52.09)

Statistical significance: ^*∗*^*P* < 0.05, ^*∗∗*^*P* < 0.01, and ^*∗∗∗*^*P* < 0.001; Student's *t*-test. Amplitudes are shown as average ± standard deviation. Stimulation: 3.0 cd s/m^2^.

**Table 2:** Summary of comparison of SD-OCT and structural findings in animal models of retinitis pigmentosa.

Animal models	Gene	Function	SD-OCT findings	EM and LM findings	References
RCS^–/–^	mertk	Phagocytosis of RPE	Hyperreflective zone in IS-OS layer	Deposition of lamellar materials	[34, 36]
			Thinning of ONL	Degeneration of IS-OS	
RHO P23H	RHO (P23H)	Phototransduction	Hyperreflective zone in IS-OS layer	Disarrangement of OS discs	[43, 44]
			Thinning of ONL	Degeneration of IS-OS	
*RHO* S334ter	RHO (S334ter)	Phototransduction	Hyperreflective zone in IS-OS layer	Degeneration of IS-OS	[47]
			Thinning of ONL	Deposition of granular materials	
RHO^–/–^	RHO	Phototransduction	Absence of EZ and IZ	Absence of OS	[33, 50–52]
			Hyporeflective zone under ELM	Short IS	
Rpe65^–/–^	Rpe65	Visual cycle	Hyperreflective zone in IS-OS layer	Degeneration of IS-OS	[35, 50]
			Thinning of ONL	Shortage of OS length	
				Lipid deposition in RPE	
rd12	Rpe65 (R44ter)	Visual cycle	Hyperreflective zone in IS-OS layer	Disorganized OS	[64]
			Thinning of ONL	Intracellular vacuoles in ONL	
				Lipid deposits in RPE	
rd 1	Pde6*β* (R560C)	Phototransduction	Extremely thin ONL		[33, 52, 70]
rd10	Pde6*β* (Y347ter)	Phototransduction	Transiently hyperreflective ONL	Intracellular vacuoles in ONL	[60, 66, 70, 71]
Arr1^–/–^	Arrestin 1	Quenching of rhodopsin		IS vesiculation	[75]
			Diffuse hyperreflective ONL by light exposure	Disruption of IS-OS junction	
				Condensation of nuclei in ONL	

IS-OS, inner and outer segments; ONL, outer nuclear layer; EZ, ellipsoid zone; IZ, interdigitation zone; RPE, retinal pigment epithelium.
